# Lead exposure as a causative factor for metabolic associated fatty liver disease (MAFLD) and a lead exposure related nomogram for MAFLD prevalence

**DOI:** 10.3389/fpubh.2022.1000403

**Published:** 2022-10-12

**Authors:** Chenyu Yang, Yuanyuan Li, Ran Ding, Huiwu Xing, Ruijue Wang, Mingman Zhang

**Affiliations:** ^1^Department of Hepatobiliary Surgery, Children's Hospital of Chongqing Medical University, Chongqing, China; ^2^Chongqing Key Laboratory of Pediatrics, Chongqing, China; ^3^National Clinical Research Center for Child Health and Disorders, Ministry of Education Key Laboratory of Child Development and Disorders, Chongqing, China; ^4^Chongqing Higher Institution Engineering Research Center of Children's Medical Big Data Intelligent Application, Chongqing, China; ^5^Center of Laboratory Medicine, Chongqing Prevention and Treatment Center for Occupational Diseases, Chongqing, China; ^6^Chongqing Key Laboratory of Prevention and Treatment for Occupational Diseases and Poisoning, Chongqing, China

**Keywords:** lead (Pb) exposure, public health, MAFLD, NHANES, fatty liver disease

## Abstract

The relationship between lead exposure and neurological disorders has been extensively studied, but the effects of lead exposure on hepatotoxicity are unknown. Metabolically related fatty liver disease (MAFLD) is an update of previous non-alcoholic fatty liver disease (NAFLD). It redefines the diagnostic conditions and emphasizes metabolic factors while considering non-alcoholic factors. Lead can affect the endocrine system and metabolism, so we believe that lead exposure may contribute to MAFLD. 41,723 individuals who had undergone blood lead testing from 2005 to 2018 in the National Health and Nutrition Examination Survey (NHANES) database were selected for this study. The characteristics of population lead exposure in the last decade or so, the effect of lead exposure on liver function and whether lead exposure can cause MAFLD were analyzed. Co-variates were adjusted according to age, ethnicity, body mass index (BMI), waist circumference, visceral adiposity index (VAI), poverty indices (PIR), diabetes, hypertension, and hyperlipidemia. The results showed that blood lead concentrations stabilized at a low level after a decreasing trend from year to year. The differences in blood lead concentrations were associated with differences in age, sex, race, education level, and PIR. Lead exposure was an independent risk factor for MAFLD, and lead and nine other factors were used as independent risk factors for MAFLD, so a nomogram was established to predict the prevalence probability of MAFLD.

## Introduction

Lead (Pb) has permanent adverse effects on the human body and lead exposure has become one of the major public health problems. In worldwide, lead exposure is result in more than one million deaths each year, and 24.4 million disability-adjusted life years (1). Lead is widely found in nature and people's lives including natural soil lead enrichment, legacy deposition, contemporary mining emissions, and lead-based paint and considered as a potent environmental toxin with its cumulative characteristics and non-biodegradable nature (2, 3). A study in China found that even if the food lead level is lower than the standard, it will eventually be enriched in the human body through cumulative effects (4). The accumulation of lead through the food chain is the main way of lead exposure, followed by direct inhalation and skin contact (4). And with age, smoking status, and alcohol consumption the blood lead levels are significantly increased (2). Although the definition of lead poisoning varies from different countries, there is a consensus that there is no exact safety threshold for lead concentration. The Centers for Disease Control and Prevention (CDC) defines a blood lead concentration >10 μg/dl as a significant increase in blood lead, while a blood lead concentration >5 μg/dl should initiate public health action (5). Blood lead level equal or >10 μg/dl is unsafe for infants, children, and women of childbearing age and blood levels equal or >30 μg/dl is unsafe for workers in occupational settings (2, 5, 6). Although, in recent decades, overall lead exposure levels have been degraded, there is still a significant lead exposure problem among poor and underserved urban populations. When considering the influence of social factors on lead exposure, it is found that economic conditions, living conditions or ethnicities also affect the concentration of blood lead (7–9).

After lead exposure, it is difficult for the human body to remove lead in a short time, so it has a cumulative effect, resulting in chronic lead poisoning. About 99% of circulating lead invades into erythrocytes and permeates into brain, liver, renal cortex, lungs, teeth, bones and other tissues and organs in varying degrees within 4–6 weeks (10). Lead is usually excreted with urine in a primordial form (inorganic lead) as well as the absorbed lead also can be secreted into bile, gastric fluid, saliva and eventually excreted through feces (2). Lead can spread all over the body and produce toxic effect, but compared with other systems, lead poisoning in nervous system has been studied most extensively. The lead toxicity mainly related to the ability to replace other bivalent cations such as Ca^2+^, Mg^2+^, Fe^2+^, and monovalent cations like Na^+^ with the form of Pb^2+^, which finally disturbs the cell biological processes including cellular signaling, protein folding, enzyme regulation, maturation, ionic transportation, oxidant-antioxidant balance, and inflammatory responses (2).

Some studies have found that lead can also affect the development of metabolism and liver disease. In fact, the liver is one of the main target organs for the accumulation of most metals after exposure (11). Lead is considered an endocrine-disrupting chemical, and in some cases, human exposure levels have been linked to the incidence of diabetes and related metabolic syndromes (5). A study based on young Mexican adults found that chronic lead exposure in early childhood was associated with high levels of liver steatosis, biomarkers, and hepatocellular damage in young adults (12). Lead may affect lipid metabolism leading to steatosis of liver cells eventually developing metabolic associated fatty liver disease (MAFLD) (13). MAFLD is an update of previous non-alcoholic fatty liver disease (NAFLD). It redefines the diagnostic conditions and emphasizes metabolic factors while considering non-alcoholic factors. The new diagnostic criteria are based on the presence of fatty liver suggested by liver biopsy histology or imaging or even blood biomarkers, while meeting one of the following three conditions: overweight/obesity, type 2 diabetes, and metabolic dysfunction (14). Studies on the pathogenesis of MAFLD have found that MAFLD originates from an underlying state of metabolic dysfunction. Therefore, to further clarify the effects of lead on liver function and whether lead could independently cause MAFLD, this project was based on the National Health and Nutrition Examination Survey (NHANES) database with sociodemographic characteristics and underlying disease information to provide guidelines for the prevention of MAFLD and better response to the public health problems of lead exposure and poisoning.

## Methods

### Database

The National Health and Nutrition Examination Survey (NHANES) is a major program of the National Center for Health Statistics (NCHS) and is responsible for providing vital health statistics for the United States. The survey is conducted annually on a representative sample of ~5,000 people in the United States. These individuals are located in counties across the country, 15 of which are visited each year. NHANES interviews included demographic, socioeconomic, dietary, and health-related questions. The examination component included medical, dental, and physiological measurements, as well as laboratory tests performed by trained medical personnel (15).

### Definition of MAFLD

The diagnosis of MAFLD is based on hepatic steatosis and requires the fulfillment of any one of overweight/obesity, diabetes, or metabolic dysfunction and according to the definition of MAFLD, metabolic dysfunction is defined as the presence of at least two of the following (14): (1) waist circumference, >102 cm for male and >88 cm for female; (2) hypertension (arterial blood pressure ≥130/85 mmHg or on antihypertensive therapy); (3) hyperlipidemia (triglycerides (TG) ≥1.70 mmol/L or on lipid-lowering therapy); (4) serum high-density cholesterol (HDL-C) levels are reduced (<1.0 mmol/L for male and <1.3 mmol/L for female); (5) diabetes or pre-diabetes, and (6) serum hypersensitivity C-reactive protein (hs-CRP) levels >2 mg/L (16).

### Data extraction

The information extracted from NHANES (year: 2005–2018) includes the following, but is not limited to, socio-demographic characteristics and lifestyle habits, such as poverty indices (PIR), smoking and alcohol consumption. Simultaneously, data on physical examination results, laboratory test results, and underlying diseases are also collected to better assess physical health, such as body mass index (BMI), hs-CRP, hypertension, and hyperlipidemia. Hypertension was confirmed either as self-reported physician-diagnosed hypertension, blood pressure >130/85 mmHg, or treatment with antihypertensive medication during survey. Hyperlipidemia was diagnosed based on the participant's health history or total cholesterol >5.72 mmol/L or triglycerides >1.70 mmol/L, and the lipid-lowering medication status. Liver dysfunction can be attributed to the abnormal values of certain enzymes and proteins such as total bilirubin (TBIL), aspartate aminotransferase (AST), alanine aminotransferase (ALT), and γ-glutamyl transpeptidase (GGT). The normal ranges for AST and ALT were ≤40 (U/L), GGT was ≤50 (U/L), and TBIL was ≤17.1 (μmol/L).

### Statistical analysis

Given the inherent limitations of the complex survey design used by NHANES (such as oversampling of certain populations, survey non-response, and post-stratification), NHANES provides weighting variables to help account for some of the biases introduced by this complex survey design (17). Thus, the weighting of data during our study improves the accuracy of the calculated estimates to be truly representative of the US civilian non-institutionalized population. The “svytable” command in the “survey” package of R is used to calculate the total number of people of different genders after weighting, and then add them together to get the total number of people that can be represented by this study. Continuous variables were expressed as $\bar{x}$ ± SD deviation. The Kolmogorov-Smirnov test was used to test whether continuous variables obeyed a normal-terms distribution. Differences between non-normally distributed continuous variables were analyzed using the Mann-Whitney *U*-test, while normally distributed variables were analyzed using the Student's *t*-test. Categorical variables were expressed as percentages and analyzed by the Chi-squared test. Weighted linear regression was used to obtain differences in blood lead concentrations between subgroups with different demographic characteristics and differences in liver function between subgroups after adjustment for co-variates. Multivariate logistic regression was used to explore independent risk factors for MAFLD. All tests were two-tailed and results with a *p*-value <0.05 were considered statistically significant. The statistical analyses described above were performed by GraphPad Prism 9 and R4.1.2.

## Results

### Exposure patterns in the US

**Figure 1:**
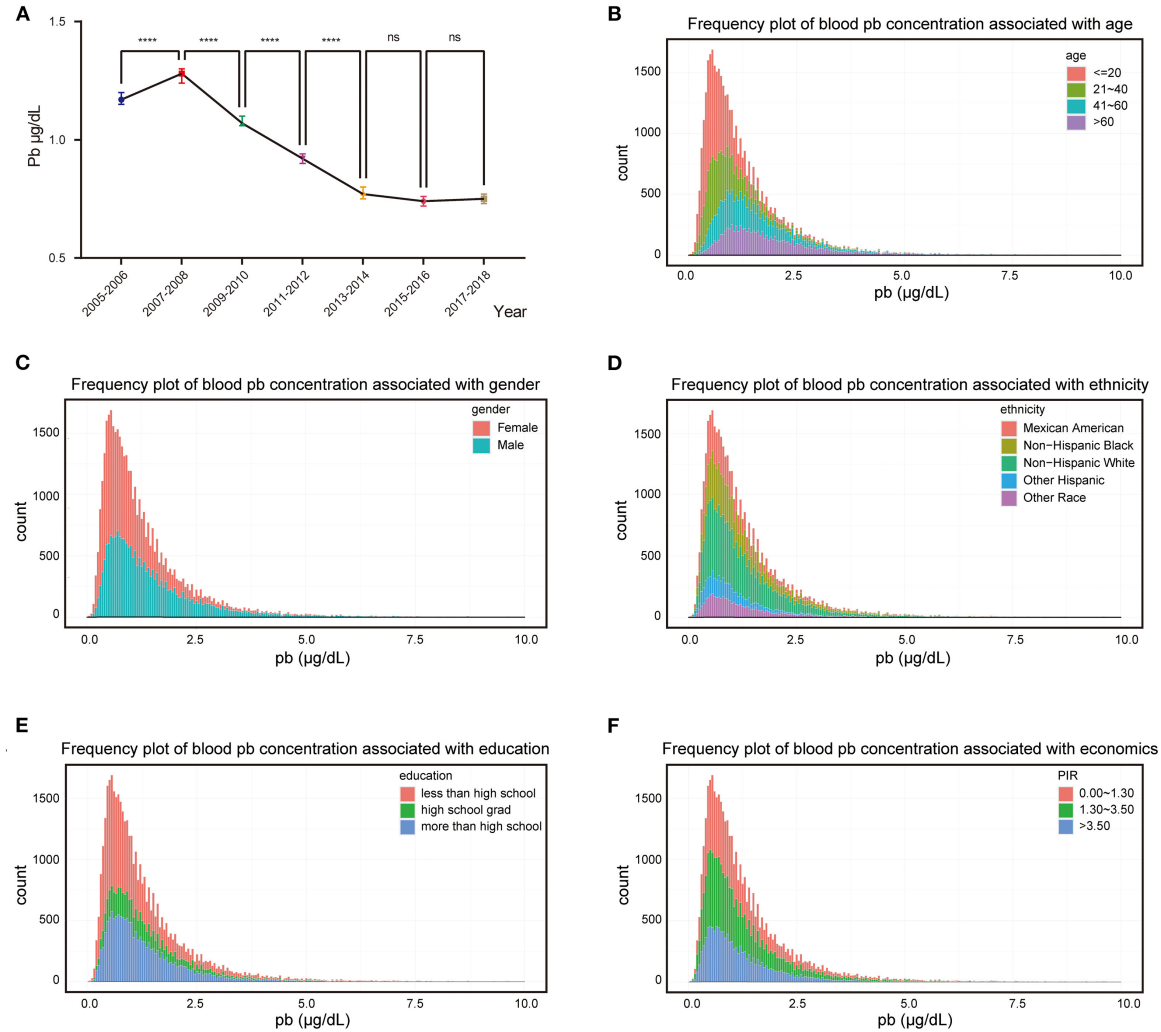
Exposure patterns in the US. **(A)** Median blood lead concentration in the population for each survey cycle, bars represent 95% confidence interval; **(B–F)** Frequency distributions of blood lead concentrations were plotted and merged with demographic characteristics. ****p-value < 0.0001; ns, no significant.

In this study, blood lead data were collected from 2005 to 2018 from 41,723 individuals with blood lead levels ranging from 0 to 61.29 μg/dl and these individuals could effectively represent 186,273,519 unsurveyed the United States residents. Analysis of population mean blood lead levels from 2005 to 2018 showed a decreasing trend in population blood lead concentrations, with blood lead levels continuing at low levels after 2013 (Figure 1A). Frequency distributions of blood lead concentrations were plotted and merged with demographic characteristics after grouping five demographic data: age, gender, ethnicity, education level, and PIR (Figures 1B–F). After grouping according to demographic characteristics, the number of people and percentage of each group and the mean blood lead concentration were shown in Table 1. We compared ethnicity-adjusted, gender-adjusted, education-adjusted, and PIR-adjusted mean blood lead concentrations for each age group by using linear regression and found that blood lead concentrations increased with age in the population. We compared the relationship between individual demographic characteristics and blood lead concentrations by linear regression, with the other four characteristics adjusted as co-variates when one characteristic was used as the independent variable. We found that all five demographic characteristics can influence the mean blood lead concentration in the population. Blood lead concentrations in the population increased with age, and men had higher mean blood lead concentrations than women. Compared to non-Hispanic white participants in the United States, blood lead concentrations were higher in non-Hispanic black participants and other race (including multi-racial) participants, lower in other Hispanic participants, no difference in blood lead between Mexican American participants and the non-Hispanic white. The level of education also affected blood lead concentration, with those with higher education having significantly lower blood lead levels than those with lower education. According to Supplemental Nutrition Assistance Program (SNAP) recommended method, we divided the population into three groups according to PIR: 0–1.3, 1.3–3.5, and ≥3.5, which represent three different family economic levels, and the higher the PIR, the better the economic condition. The blood lead concentration in the population showed a negative correlation with the economic level, the worse the economic condition of the family, the higher the blood lead concentration (Table 2).

**Table 1:** Mean blood lead concentration for age, gender, ethnic, education, and PIR group.

**Objects**	**Count** **(percent)**	**Blood lead** **(μg/dl, $\bar{X}$ ± SD)**
**Age**		
≤20	13,962 (34.46%)	0.80 ± 0.01
21–40	9,640 (23.10%)	1.06 ± 0.02
41–60	9,295 (22.27%)	1.56 ± 0.03
>60	8,826 (21.15%)	1.87 ± 0.03
**Gender**		
Female	20,572 (49.29%)	1.11 ± 0.01
Male	21,151 (50.68%)	1.52 ± 0.02
**Ethnicity**		
Non-Hispanic white	16,397 (39.29%)	1.30 ± 0.02
Mexican American	7,696 (18.44%)	1.31 ± 0.04
Non-Hispanic black	9,405 (22.54%)	1.41 ± 0.03
Other Hispanic	3,772 (9.04%)	1.11 ± 0.04
Other race—including multi-racial	4,453 (10.67%)	1.36 ± 0.04
**Education**		
Less than high school	19,356 (46.38%)	1.25 ± 0.02
High school grad	7,133 (17.09%)	1.48 ± 0.03
More than high school	15,234 (36.50%)	1.28 ± 0.02
**PIR**		
0.00–1.30	14,824 (35.52%)	1.41 ± 0.03
1.30–3.50	15,554 (37.27%)	1.31 ± 0.02
>3.50	11,345 (27.19%)	1.25 ± 0.02

**Table 2:** Differences of blood lead concentration in different subgroups by adjusting demographic characteristics.

**Objects**	**Differences in blood lead concentration (μg/dl)**	***p*** **-value**
**Age**		
≤20	Reference^#^	–
21–40	0.55	1.96e−29
41–60	1.09	3.89e−47
>60	1.38	8.60e−67
**Gender**		
Female	Reference^$^	–
Male	0.46	< 2e−16
**Ethnicity**		
Non-Hispanic white	Reference^θ^	–
Mexican American	0.08	0.10449
Non-Hispanic black	0.16	9.27e−06
Other Hispanic	−0.13	0.00173
Other race—including multi-racial	0.16	3.84e−05
**Education**		
Less than high school	Reference^Φ^	–
High school grad	−0.20	6.80e−11
More than high school	−0.32	< 2e−16
**PIR**		
0.00–1.30	Reference^Ψ^	–
1.30–3.50	−0.18	3.42e−08
>3.50	−0.24	3.19e−09

# After adjusting for sex, ethnicity, education level, and PIR, compared with the age ≤ 20 years group, the average blood lead concentration was 0.55 μg/dl higher in the 21–40 years group, 1.09 μg/dl higher in the 41–60 years group, and 1.38 μg/dl higher in the >60 years group.

$ After adjusting for age, ethnicity, education level, and PIR, the average blood lead concentration in male is 0.1 g higher than that in female.

θ After adjusting for age, sex, education level, and PIR, compared to Non-Hispanic white population, the average blood lead concentration was not significantly different in Mexican American population, 0.16 μg/dl higher in Non-Hispanic black population, 0.13 μg/dl lower in Other Hispanic population, and 0.16 μg/dl higher in other race.

Φ After adjusting for age, sex, ethnicity, and PIR, compared to those whose educational level was less than high school, the mean blood lead concentration was reduced by 0.20 μg/dl in the high school grad group and 0.32 μg/dl in the more than high school group.

Ψ After adjusting for age, sex, ethnicity, and education level, compared with the PIR0.00–1.30 group, the average blood lead concentration in the PIR1.30–3.50 group decreased by 0.18 g, and the average blood lead concentration in the PIR >3.50 group decreased by 0.24 g.

### Lead could induce liver dysfunction

**Figure 2:**
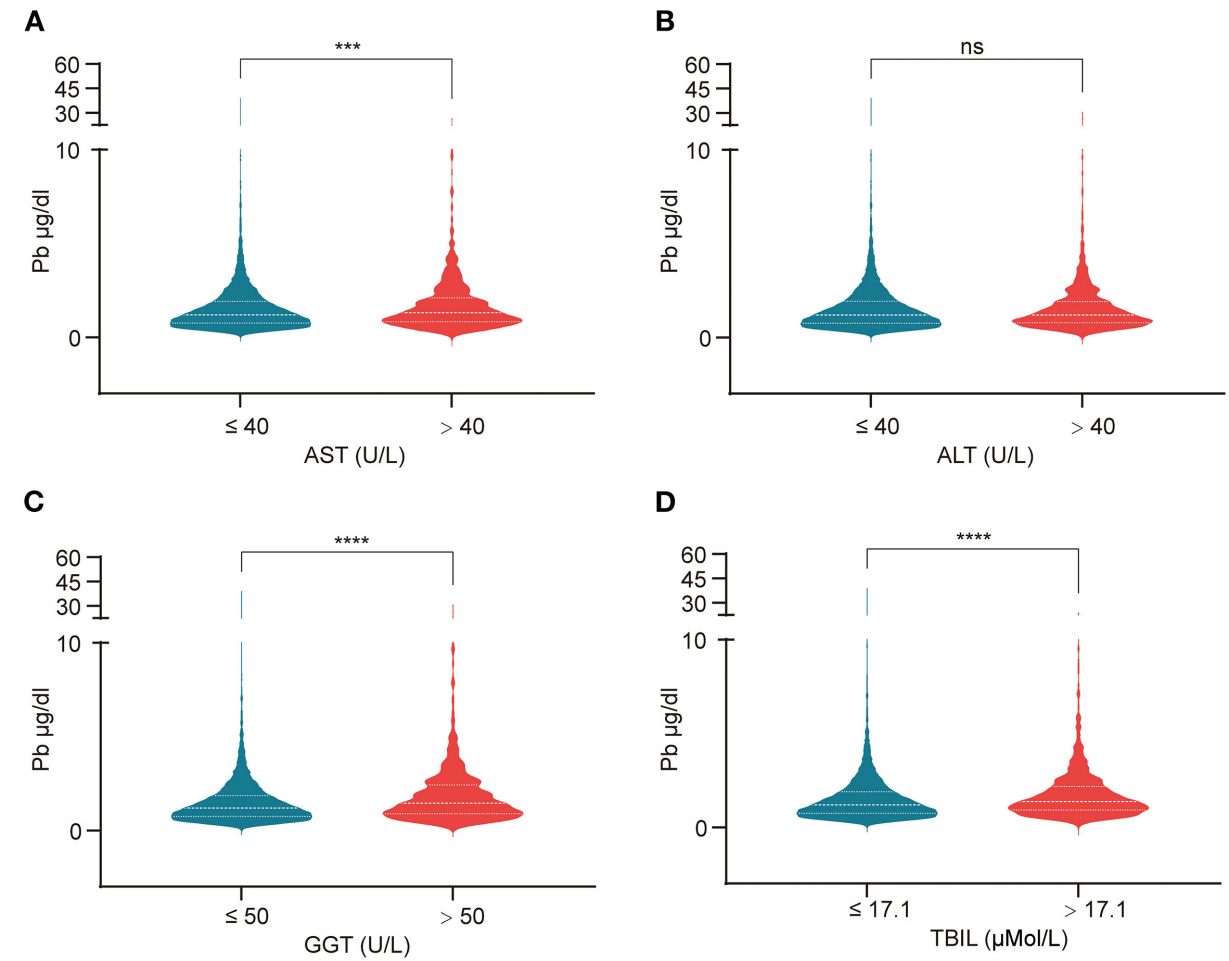
The violin plots reflect the effect of lead exposure on liver function. **(A)** AST > 40 U/L group had higher blood lead concentration; **(B)** The effect of lead exposure on ALT was not significant; **(C)** GGT > 50 U/L group had higher blood lead concentration; **(D)** TBIL > 17.1 μMol/L group had higher blood lead concentration. ***p-value < 0.001; ****p-value < 0.0001; ns, no significant.

**Figure 3:**
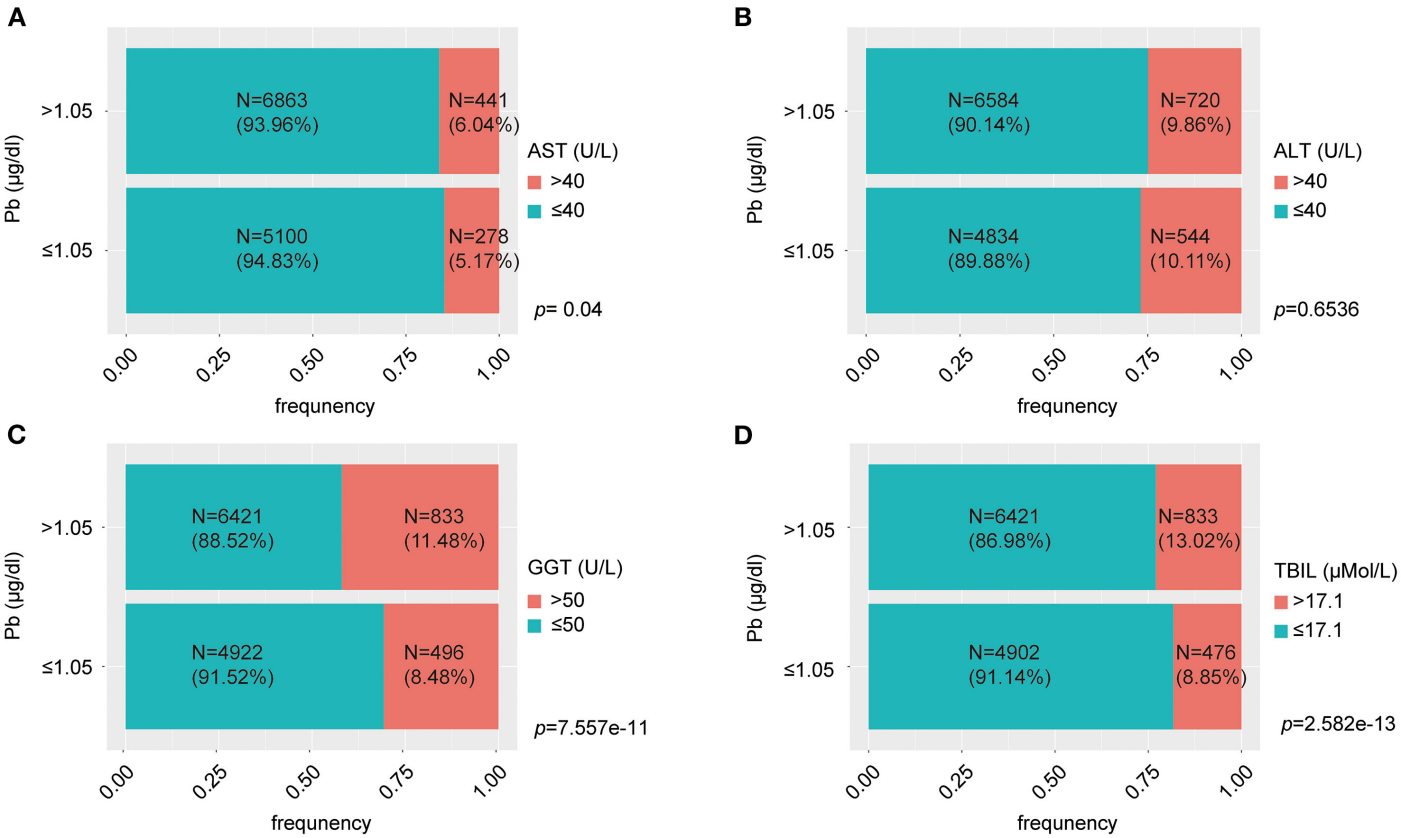
The percent bar charts reflect proportion of normal and abnormal liver function at different levels of lead exposure. **(A)** The group with blood lead concentration >1.5 μg/dl had a greater proportion of individuals with AST > 40 U/L; **(B)** There was no significant difference in the proportion of individuals with ALT > 40 U/L between the high and low blood lead concentration groups; **(C)** The group with blood lead concentration >1.5 μg/dl had a greater proportion of individuals with GGT > 50 U/L; **(D)** The group with blood lead concentration > 1.5 μg/dl had a greater proportion of individuals with TBIL > 17.1 μMol/L.

We evaluated the effect of lead on liver function. Liver function was assessed by liver enzymatic parameters and total bilirubin. We obtained liver function data and information on smoking, alcohol consumption, hypertension, hyperlipidemia, diabetes, metabolic syndrome, height, weight, body mass index (BMI) and visceral adiposity index (VAI) from the 41,723 people mentioned above. After the exclusion of individuals with missing values, the remaining 12,682 individuals were included in the subsequent study. These individuals could effectively represent 67,519,958 unsurveyed the United States residents. The population was divided into normal and abnormal groups according to the normal values of ALT, AST, GGT and TBIL, and whether there were differences in blood lead concentrations between the two groups were compared. We found that the median blood lead concentrations in the ALT, GGT, and TBIL abnormal groups were higher than those in the normal group, while there was no significant difference between the AST abnormal group and the normal group (Figure 2). Subsequently, we divided the population into two groups according to the median blood lead concentration <1.05 and ≥1.05 μg/dl, and further distinguished whether the liver function was normal in the two groups separately. Elevated blood lead concentrations were found to be more likely to lead to liver dysfunction, and more people in the high blood lead concentration group had AST, GGT, and TBIL outside the normal range compared to the low blood lead concentration group, but there was no difference in the proportion of abnormal ALT between the two groups (Figure 3). We included diabetes, hypertension, hyperlipidemia, BMI, VAI, which were considered to affect liver function in clinical work, and demographic data as co-variates. Weighted linear regression was used to determine the specific differences of liver function between high and low blood lead groups. Compared to the lead <1.05 μg/dl group, the lead ≥1.05 μg/dl group had an increase of 1.63 U/L in ALT, 1.42 U/L in AST, 3.42 U/L in GGT, and 1.20 μMol/L in TBIL (Table 3).

**Table 3:** Differences of liver function between high and low blood lead groups.

**Liver function**	**Differences of liver function (compared with blood lead concentration <1.05 μg/dl group)**	***p*** **-value**
ALT (U/L)	1.63^Φ^	3.79e−05
AST (U/L)	1.42^Ψ^	4.34e−05
GGT (U/L)	3.42^$^	0.000266
TBIL (μMol/L)	1.20^#^	8.80e−11

Φ Compared to the population with blood lead concentration <1.05 μg/dl, the mean serum ALT concentration was increased by 1.63 U/L in the population with blood lead concentration ≥1.05 μg/dl.

Ψ Compared to the population with blood lead concentration <1.05 μg/dl, the mean serum AST concentration was increased by 1.42 U/L in the population with blood lead concentration ≥1.05 μg/dl.

$ Compared to the population with blood lead concentration <1.05 μg/dl, the mean serum GGT concentration was increased by 3.42 U/L in the population with blood lead concentration ≥1.05 μg/dl.

# Compared to the population with blood lead concentration <1.05 μg/dl, the mean serum TBIL concentration was increased by 1.20 μMol/L in the population with blood lead concentration ≥1.05 μg/dl.

### Diagnosis of MAFLD

Lead can affect metabolism, and it has also been demonstrated that lead could affect liver function, so we hypothesized that lead may induce MAFLD. We extracted information on liver steatosis, metabolic dysfunction, triglyceride, and hs-CRP from the 12,682 individuals with liver function data described above. Of these, 10,654 individuals did not enter the subsequent study because they lacked the necessary information to diagnose MAFLD. The remaining 2,028 individuals could effectively represent 12,015,077 unsurveyed the United States residents. 1,067 people were identified as having MAFLD and 961 were not (Supplementary Table S1).

### Lead affects the development of MAFLD

**Figure 4:**
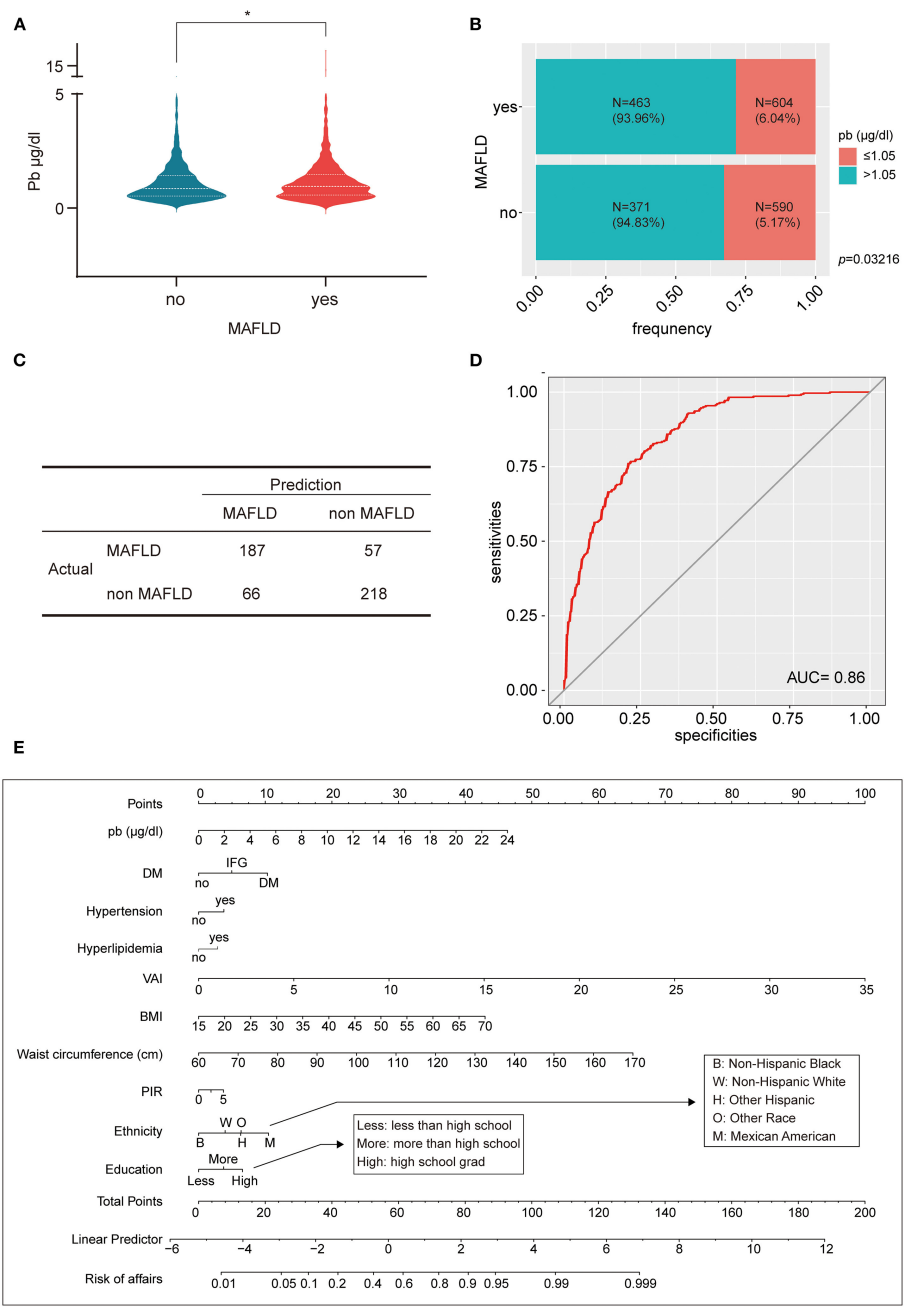
Lead affects the development of MAFLD. **(A)** The violin plot reflects that MAFLD had higher blood lead concentration than non-MAFLD; **(B)** The percent bar chart reflects that the MAFLD group a greater proportion of individuals with blood lead concentration >1.5 μg/dl; **(C)** The four-grid table shows the difference between the predicted value and the actual value of the multifactorial logistic regression model in the validation set. **(D)** The ROC curve evaluated the effectiveness of multifactorial logistic regression model, AUC = 0.86; **(E)** A lead exposure related nomogram for MAFLD prevalence. *p-value < 0.05.

The above 2,028 people were divided into MAFLD diseased and non-diseased groups, and the lead concentration was significantly higher in the diseased group than in the non-diseased group (Figure 4A). The two groups were further divided into two subgroups: blood lead <1.05 and ≥1.05 μg/dl. The proportion of blood lead ≥1.05 μg/dl in MAFLD group was 1.16 times higher than that in non-MAFLD group (Figure 4B). To further investigate whether lead is a pathogenic mechanism in MAFLD, 1,500 individuals were randomly selected from 2,028 as the training set and 528 as the validation set. Lead, diabetes, smoking, hypertension, hyperlipidemia, VAI, metabolic syndrome, BMI, waist circumference, PIR, age, gender, ethnicity, and education level were included in the logistic regression and were performed in the training set. After stepwise regression, lead, DM, hypertension, hyperlipidemia, VAI, BMI, waist circumference, PIR, ethnicity, and education level were identified as independent risk factors for MAFLD and the regression model was refitted, and the co-efficients was summarized in Tables 1–3. The multifactorial logistic regression model was validated in the validation set, with the model predicting MAFLD with an accuracy of 76.76% and negating MAFLD with an accuracy of 76.63%, giving the model an overall efficiency of 76.70% (Figure 4C). The efficacy of the model was further assessed using the ROC curve with AUC = 0.86, indicating that the logistic regression model has good discrimination (Figure 4D). Finally, all 2,028 subjects were included in the logistic regression model, and a nomogram was created to visualize the model, enabling visualization of the probability of lead exposure with other different causative factors leading to MAFLD (Figure 4E). For a given patient, each independent risk factor had a specific value. The score for each indicator was obtained by projecting the values onto the first row of the “Points” scale. The scores for all independent risk factors were simply summed to obtain a total score. The final score was found in “Total points” and projected to “Risk of affairs” to find the probability of developing MAFLD after lead exposure for the patient (Supplementary Figure S1).

## Discussion

Our study is the first to identify lead exposure as an independent risk factor for MAFLD in a population, which can cause hepatic insufficiency at blood lead concentrations <5 μg/dl, resulting in elevated AST, GGT, and TBIL. Based on the results of the study, we developed a nomogram that facilitates the clinical diagnosis of MAFLD.

The toxicity of lead was widely recognized in the late 19th century as a neurotoxin accumulated in soft tissues and bones; it destroys the nervous system, interferes with the function of biological enzymes, and leads to nervous system disorders, from behavioral problems to brain damage. It also affects general health, cardiovascular and renal systems (2). In recent decades, there has been a marked decline in blood lead levels in the US population, largely due to the introduction of unleaded gasoline in the 1980s. This change did have a rapid impact on overall lead exposure levels in children, with average blood lead levels decreasing from 13.7 3.2 μg/dl between 1976 and 1994 (5). The CDC estimated that the average blood lead level in the US population would be reduced to 1.5 μg/dl in 2005 (18), and our results confirm this prediction and find that it has fallen further and remained at a lower level (0.5–1 μg/dl) in the following decade (Figure 1A). A notable feature of the current lead exposure situation in the United States is that it is unevenly distributed. Exposure levels are higher among poor, underserved populations, primarily in urban industrial areas (5). Similar results were found when we grouped the population by PIR for economic conditions and found that blood lead concentrations were significantly lower in those with good economic conditions compared to those with poor economic conditions. The mean blood lead concentrations in the PIR 1.30–3.50 and PIR >3.50 groups were lower than those in the PIR 0.00–1.30 group by 0.18 μg/dl and 0.24 μg/dl, respectively. We also found that the average blood lead concentration increased with age, which may be related to the bioaccumulation effect of lead. Of course, it also had something to do with the social environment of different groups, which may give older people more exposure to lead than the younger group. For example, older adults are more likely than younger groups to have had greater exposure to leaded gasoline or to use leaded alloys that accumulated in their bodies prior to the policy change. Blood lead levels also differed between genders, with males having higher mean blood lead levels than females at 0.46 μg/dl. Studies have shown that women and men are exposed to the same levels of lead exposure (19), with men having higher blood lead levels and possibly being more susceptible to the effects of lead poisoning than women (20). This result suggested that the minimum intervention blood lead concentrations for males and females may need to be defined separately. There were significant differences in blood lead levels between different ethnicities, with Mexican Americans having no significant difference in blood lead levels compared to Non-Hispanic White, Other Hispanic having lower blood lead levels than Non-Hispanic White, and Non-Hispanic Black and Other Race (Including Multi-Racial) having higher blood lead levels than Non-Hispanic White. The level of education also affects blood lead levels, with the higher the level of education the lower the blood lead levels. It may be that people with higher levels of education have a better sense of precaution or a better economic base and living conditions that reduce the chances of lead exposure in their lives.

We assessed liver injury from lead exposure by liver function, which includes liver enzymes (AST, ALT, and GGT) and TBIL. Blood lead concentrations were elevated in those with abnormal AST, GGT, and total bilirubin compared to the normal population, with no significant differences observed in AST. When the population was grouped according to median blood lead levels, the proportion of those with abnormal AST, GGT, and TBIL was significantly higher in the high concentration group than in the low concentration group, with the same results for ALT. After including 12 co-variates, an increase of 1.63 U/L in ALT, 1.42 U/L in AST, 3.42 U/L in GGT, and 1.20 μMol/L in TBIL was found compared to the low blood lead concentration group. This suggests that the currently defined normal range of blood lead concentrations may be relatively loose and dangerous, as organ damage can occur even below 5 μg/dl. The mechanism of lead-induced liver damage is not fully understood. Oxidative stress is thought to be the main mechanism of lead poisoning. Lead may cause damage to the liver by inducing oxidative stress and oxidative damage to cellular lipids, proteins and DNA and inflammatory markers in the liver (21). We therefore hypothesized that lead may cause metabolic disorders in the liver while affecting liver function.

Metabolic (dysfunction) associated fatty liver disease (MAFLD) is a newly defined fatty liver disease that has recently been used as an alternative to NAFLD to better define the cause of fatty liver and to guide clinical trial design and drug development (14). The heterogeneity of the clinical presentation and course of MAFLD may be influenced by a variety of factors, including age, gender, hormonal status, ethnicity, diet, alcohol consumption, smoking, genetic predisposition, microbiota, metabolic status and superimposed disease states (14). One of the most intuitive results was the significantly higher blood lead levels in the MAFLD group compared to the non-MAFLD group. Also, the number of MAFLD was greater in the high blood lead concentration group. These were the basis for further research. We used logistic regression model to demonstrate that lead was an independent risk factor for MAFLD along with DM, hypertension, hyperlipidemia, VAI, BMI, waist circumference, PIR, ethnicity, and education level. The prevalence of MAFLD, the risk of complications and the likelihood of disease-specific mortality increase with age. Gender differences also appear to contribute to differences in prevalence or outcomes in MAFLD. But we did not find age and gender to be independent risk factors for MAFLD in this study. Ten independent risk factors of MAFLD were used to draw a nomogram. Ten independent risk factors for MAFLD were used to develop a nomogram to assess the combined effect of multiple causative factors on the risk of developing MAFLD. The 10 independent risk factors of lead exposure patients were scored one by one, and each risk factor corresponds to a score in the first row of the “Points” scale. The scores were summed up to get “total point” and the corresponding relationship between “total point” and “risk of affairs” was determined to predict the probability of MAFLD in lead exposure patients. According to current lead exposure standards, there is a 1–5% probability of developing MAFLD with a blood lead concentration of >5 or >10 μg/dl only. DM, hypertension, and hyperlipidemia could affect metabolism and therefore have an impact on the development of MAFLD. The nomogram showed that DM had the greatest impact of the three, and even IFG alone can promote the progression of MAFLD. Obesity is a recognized risk factor for MAFLD, but it may be more meaningful to distinguish between different types of obesity rather than a single BMI to analyze the effect of obesity on MAFLD. A greater amount of visceral adipose tissue relative to peripheral and subcutaneous adipose tissue is associated with greater metabolic risk (14). We similarly found VAI and waist circumference to be superior in predicting MAFLD compared to BMI. Before MAFLD was defined, a meta-analysis found that the prevalence of NAFLD was highest in Hispanics, lowest in blacks and intermediate in whites (22). Our results including more ethnicities indicated that Non-Hispanic Black had the least impact on MAFLD, Mexican American had the most impact on MAFLD, with Non-Hispanic White, Other Hispanic and Other Race (Including Multi-Racial) ranked in the middle in order of risk from small to large. Poverty and low educational attainment did not have a significant impact on MAFLD, and people with good economic conditions and high educational attainment may instead be more likely to develop MAFLD, a phenomenon that was the opposite of lead exposure. Our nomogram items are routine clinical variables readily available to clinicians, thus allowing the nomogram to be easily adopted in practice, even by patients themselves, to determine their condition. At the same time this nomogram can prospectively and dynamically assess the high green size of MAFLD occurrence as the patient's own condition changes. Our nomogram items are routine clinical variables readily available to clinicians, thus allowing the nomogram to be easily adopted in practice. Admittedly, many clinicians will not use blood lead testing as a preferred laboratory test, leading to possible limitations in the clinical practice of this nomogram. However, lead exposure remains a thorny public health problem worldwide, and the health outcomes for the large number of lead-exposed patients that have now accumulated should be taken seriously. We hope that this study will draw the attention of clinicians to protect liver function and prevent subsequent liver disease in patients with lead exposure or acute and chronic lead poisoning while paying attention to the protection of the central nervous system. This nomogram may also provide assistance in prospectively assessing dynamically the prevention of MAFLD after lead exposure.

Our study was a cross-sectional study and although it was established that lead exposure can cause MAFLD, the relationship between lead exposure duration and MAFLD was not well-studied. Subsequent cohort studies will be needed to examine the quantitative and temporal effects of lead exposure in relation to MAFLD.

## Conclusion

Although the average blood lead concentration in the population has been decreasing each year for the last decade as sources of lead exposure have been controlled, it has remained stable at a low level for the last few years. After adjusting for demographic characteristics such as economic, educational, and ethnic characteristics, there are significant differences in mean blood lead concentrations between population subgroups. Therefore, the prevention and control of lead exposure should not be neglected. Our study identified lead as a pathogen of MAFLD and that blood lead concentrations <5 μg/dl (recommended reference levels for initiating public health actions) could cause MAFLD, further suggesting that there is no safe threshold for lead exposure. This study provides valid evidence for the study and prevention of lead exposure to metabolic diseases.

## Data availability statement

Publicly available datasets were analyzed in this study. This data can be found at: https://wwwn.cdc.gov/nchs/nhanes/Default.aspx.

## Author contributions

CY: conceptualization, methodology, data curation, formal analysis, writing—original draft, writing—review and editing, and project administration. YL: methodology, data curation, formal analysis, writing—original draft, writing—review and editing, and project administration. RD: methodology and writing—review and editing. HX: methodology and formal analysis. RW and MZ: conceptualization, writing—review and editing, and project administration. All authors contributed to the article and approved the submitted version.

## Conflict of interest

The authors declare that the research was conducted in the absence of any commercial or financial relationships that could be construed as a potential conflict of interest.

## Publisher's note

All claims expressed in this article are solely those of the authors and do not necessarily represent those of their affiliated organizations, or those of the publisher, the editors and the reviewers. Any product that may be evaluated in this article, or claim that may be made by its manufacturer, is not guaranteed or endorsed by the publisher.
