# Supplementary material for: Lead exposure as a causative factor for metabolic associated fatty liver disease (MAFLD) and a lead exposure related nomogram for MAFLD prevalence
Source: Front Public Health. 2022 Oct 12;10:1000403. doi: 10.3389/fpubh.2022.1000403 (PMC9597460; doi:10.3389/fpubh.2022.1000403)
Supplement: Supplementary file 1 [file Table_1.DOCX]

**Table.1s Liver function data related to blood lead concentration in groups with different demographic characteristics**

|  | **ALT (U/L)** | | **AST (U/L)** | | **GGT (U/L)** | | **TBIL (μmol/l)** | |
| --- | --- | --- | --- | --- | --- | --- | --- | --- |
|  | **≤1.05** | **>1.05** | **≤1.05** | **>1.05** | **≤1.05** | **>1.05** | **≤1.05** | **>1.05** |
| **Ethnicity** |  |  |  |  |  |  |  |  |
| Mexican American | 28.55  (26.34,30.75) | 32.92  (31.08,34.75) | 23.67  (23.15,24.19) | 26.00  (25.34,26.65) | 23.94  (23.03,24.85) | 29.61  (28.07,31.15) | 11.55  (11.28,11.83) | 13.18  (12.90,13.45) |
| Non-Hispanic Black | 20.85  (19.94,21.75) | 24.23  (23.09,25.38) | 26.02  (23.90,28.14) | 28.21  (26.93,29.48) | 27.78  (25.13,30.42) | 39.36  (35.51,43.20) | 11.18  (10.63,11.74) | 12.77  (12.38,13.17) |
| Non-Hispanic White | 24.28  (23.62,24.95) | 25.63  (24.87,26.39) | 22.74  (21.98,23.50) | 27.36  (25.64,29.08) | 25.34  (23.98,26.70) | 38.25  (34.35,42.16) | 10.35  (9.88,10.82) | 11.82  (11.46,12.18) |
| Other Hispanic | 27.08  (25.38,28.78) | 28.29  (26.24,30.35) | 25.58  (23.43,27.74) | 26.01  (24.66,27.36) | 27.77  (25.19,30.34) | 35.55  (31.26,39.85) | 10.87  (10.42,11.31) | 11.95  (11.45,12.46) |
| Other Race  (Including Multi-Racial) | 23.21  (21.54,24.88) | 25.16  (23.81,26.50) | 23.39  (22.02,24.77) | 25.04  (23.82,26.26) | 24.75  (19.58,29.92) | 28.87  (26.00,31.74) | 11.00  (10.32,11.68) | 12.67  (12.07,13.27) |
| **Gender** |  |  |  |  |  |  |  |  |
| Male | 30.26  (29.29,31.24) | 29.38  (28.43,30.33) | 26.28  (25.59,26.98) | 27.74  (26.96,28.53) | 30.55  (28.80,32.30) | 34.88  (32.92,36.83) | 12.83  (12.55,13.12) | 14.05  (13.77,14.33) |
| Female | 20.74  (20.21,21.27) | 21.48  (20.89,22.08) | 22.38  (21.85,22.91) | 24.09  (23.59,24.59) | 21.04  (20.22,21.85) | 26.53  (24.95,28.12) | 10.36  (10.11,10.60) | 11.31  (11.09,11.54) |
| **Age** |  |  |  |  |  |  |  |  |
| 18~34 | 24.30  (23.42,25.18) | 28.10  (26.51,29.69) | 23.59  (22.84,24.34) | 25.94  (24.59,27.30) | 21.33  (20.25,22.42) | 25.64  (23.98,27.30) | 11.40  (11.05,11.75) | 13.41  (12.86,13.95) |
| 34~49 | 24.78  (24.01,25.56) | 28.56  (27.41,29.71) | 23.93  (23.24,24.62) | 26.68  (25.74,27.61) | 25.88  (24.55,27.21) | 35.54  (31.35,39.72) | 11.43  (11.08,11.78) | 12.69  (12.31,13.06) |
| 49~63 | 25.62  (24.38,26.86) | 27.21  (26.23,28.20) | 24.65  (23.66,25.63) | 27.18  (26.26,28.09) | 28.63  (26.29,30.96) | 33.73  (31.95,35.50) | 11.06  (10.62,11.50) | 12.92  (12.51,13.32) |
| ≥63 | 22.06  (21.20,22.93) | 21.84  (21.13,22.55) | 23.73  (22.93,24.53) | 24.90  (24.29,25.52) | 27.20  (24.11,30.29) | 28.04  (26.66,29.42) | 11.08  (10.46,11.71) | 12.88  (12.62,13.14) |
| **PIR** |  |  |  |  |  |  |  |  |
| 0.00~1.30 | 25.48  (24.26,26.70) | 25.27  (24.45,26.10) | 24.42  (23.36,25.49) | 25.97  (25.21,26.74) | 27.21  (25.68,28.74) | 34.58  (32.40,36.76) | 10.40  (10.09,10.72) | 11.90  (11.58,12.23) |
| 1.30~3.50 | 24.37  (23.56,25.19) | 26.09  (25.00,27.17) | 23.63  (22.96,24.29) | 26.25  (25.38,27.12) | 24.53  (23.34,25.72) | 32.17  (29.47,34.87) | 11.14  (10.82,11.46) | 12.82  (12.51,13.14) |
| >3.50 | 24.03  (23.18,24.87) | 26.56  (25.62,27.50) | 23.89  (23.21,24.57) | 26.34  (25.60,27.09) | 23.77  (22.07,25.46) | 29.10  (27.22,30.99) | 11.89  (11.57,12.22) | 13.53  (13.20,13.86) |
| **DM** |  |  |  |  |  |  |  |  |
| NO | 22.54  (22.05,23.04) | 25.41  (24.72,26.09) | 23.06  (22.57,23.56) | 25.73  (25.13,26.33) | 21.56  (20.67,22.44) | 27.67  (26.59,28.74) | 11.44  (11.19,11.70) | 12.90  (12.65,13.14) |
| DM | 28.68  (27.27,30.09) | 27.08  (25.92,28.24) | 26.30  (24.76,27.84) | 26.75  (25.90,27.59) | 35.89  (32.40,39.39) | 41.23  (35.65,46.82) | 10.93  (10.54,11.33) | 12.83  (12.46,13.21) |
| IFG | 30.11  (27.02,33.19) | 27.77  (26.20,29.34) | 25.74  (23.74,27.73) | 26.05  (24.87,27.24) | 29.88  (27.16,32.60) | 34.85  (30.70,39.00) | 10.34  (9.71,10.96) | 12.54  (12.09,12.99) |
| IGT | 28.34  (25.48,31.20) | 27.46  (25.75,29.17) | 25.20  (23.94,26.46) | 29.35  (27.37,31.33) | 27.30  (24.74,29.85) | 36.62  (31.99,41.24) | 12.25  (11.57,12.92) | 13.75  (13.25,14.26) |
| **Smoke** |  |  |  |  |  |  |  |  |
| Never | 24.17  (23.52,24.82) | 25.50  (24.90,26.10) | 23.93  (23.38,24.47) | 25.46  (24.92,25.99) | 23.09  (22.24,23.94) | 27.54  (26.14,28.95) | 11.58  (11.33,11.83) | 13.38  (13.14,13.62) |
| Former | 24.93  (23.69,26.18) | 26.33  (25.33,27.33) | 23.92  (22.92,24.92) | 26.37  (25.61,27.13) | 27.20  (24.92,29.48) | 30.95  (29.27,32.63) | 11.10  (10.69,11.50) | 13.28  (12.93,13.64) |
| Now | 25.01  (23.67,26.36) | 26.91  (25.51,28.32) | 23.73  (22.59,24.87) | 27.41  (26.06,28.77) | 28.84  (25.15,32.54) | 38.71  (34.42,43.00) | 10.39  (9.94,10.85) | 11.70  (11.33,12.06) |
| **Hypertension** |  |  |  |  |  |  |  |  |
| No | 23.07  (22.55,23.59) | 25.97  (25.21,26.74) | 23.08  (22.70,23.46) | 25.69  (25.02,26.36) | 21.36  (20.68,22.05) | 27.50  (26.29,28.71) | 11.44  (11.17,11.70) | 13.09  (12.81,13.37) |
| Yes | 27.72  (26.50,28.93) | 26.28  (25.33,27.23) | 25.84  (24.70,26.99) | 26.91  (26.11,27.71) | 32.76  (30.20,35.32) | 36.36  (33.82,38.89) | 11.04  (10.68,11.39) | 12.70  (12.45,12.94) |
| **Hyperlipidemia** |  |  |  |  |  |  |  |  |
| No | 21.36  (20.64,22.09) | 25.29  (23.44,27.13) | 22.72  (21.99,23.45) | 26.49  (24.92,28.05) | 20.05  (18.77,21.32) | 25.70  (23.51,27.88) | 11.71  (11.37,12.04) | 13.13  (12.66,13.60) |
| Yes | 26.11  (25.51,26.70) | 26.36  (25.88,26.85) | 24.54  (24.03,25.04) | 26.15  (25.63,26.66) | 27.27  (26.16,28.37) | 33.21  (31.59,34.83) | 11.11  (10.84,11.37) | 12.85  (12.64,13.06) |
| **VAI** |  |  |  |  |  |  |  |  |
| Q1 (0.089,0.868) | 20.47  (19.66,21.28) | 24.58  (23.10,26.06) | 23.19  (22.25,24.13) | 27.07  (25.86,28.28) | 18.69  (17.65,19.73) | 26.64  (24.80,28.48) | 11.81  (11.38,12.24) | 13.21  (12.80,13.63) |
| Q2 (0.868,1.422) | 22.57  (21.39,23.75) | 24.70  (23.77,25.63) | 22.80  (22.06,23.55) | 26.26  (25.22,27.29) | 22.16  (20.47,23.85) | 27.93  (25.94,29.92) | 11.60  (11.22,11.97) | 13.27  (12.86,13.68) |
| Q3 (1.422,2.409) | 25.08  (24.24,25.93) | 26.19  (25.22,27.15) | 23.72  (23.02,24.41) | 25.03  (24.35,25.72) | 25.10  (23.42,26.78) | 29.49  (27.85,31.13) | 10.96  (10.61,11.32) | 12.69  (12.38,13.01) |
| Q4 (2.409,71.3106) | 30.11  (29.00,31.23) | 28.88  (27.94,29.81) | 25.96  (25.02,26.89) | 26.62  (25.76,27.49) | 33.65  (31.44,35.86) | 41.41  (37.08,45.74) | 10.83  (10.49,11.16) | 12.51  (12.22,12.80) |
| **MetS** |  |  |  |  |  |  |  |  |
| No | 23.35  (22.86,23.85) | 25.59  (24.97,26.21) | 23.43  (22.97,23.89) | 26.07  (25.52,26.62) | 22.86  (22.08,23.65) | 29.78  (28.46,31.10) | 11.43  (11.19,11.66) | 12.98  (12.75,13.22) |
| Yes | 32.34  (30.48,34.20) | 29.81  (28.53,31.08) | 27.32  (26.10,28.53) | 27.33  (26.28,28.37) | 38.29  (34.26,42.33) | 43.16  (38.79,47.52) | 10.54  (10.03,11.06) | 12.44  (11.97,12.91) |
| **Waist (cm)** |  |  |  |  |  |  |  |  |
| Q1 (61.1,87.4) | 19.01  (18.28,19.74) | 22.26  (21.01,23.50) | 22.34  (21.65,23.04) | 26.06  (25.09,27.03) | 16.94  (15.96,17.92) | 25.48  (23.32,27.65) | 12.04  (11.67,12.40) | 13.08  (12.70,13.46) |
| Q2 (87.4,97.7) | 23.04  (22.03,24.04) | 25.53  (24.40,26.66) | 23.28  (22.54,24.01) | 26.03  (25.02,27.04) | 21.63  (20.20,23.06) | 29.93  (28.26,31.61) | 11.89  (11.45,12.33) | 12.79  (12.47,13.12) |
| Q3 (97.7,108.7) | 25.93  (25.02,26.83) | 28.20  (27.20,29.19) | 23.70  (23.03,24.37) | 27.09  (26.05,28.12) | 27.03  (25.42,28.64) | 33.60  (31.58,35.62) | 11.03  (10.65,11.42) | 13.31  (12.95,13.68) |
| Q4 (108.7,176.0) | 30.17  (28.79,31.55) | 28.19  (27.23,29.16) | 26.23  (25.02,27.44) | 25.69  (25.06,26.32) | 33.65  (31.55,35.75) | 36.44  (32.34,40.54) | 10.34  (9.99,10.69) | 12.47  (12.10,12.85) |
| **Height (cm)** |  |  |  |  |  |  |  |  |
| Q1 (137.9,160.2) | 21.66  (20.65,22.67) | 21.99  (21.15,22.83) | 23.27  (22.07,24.46) | 24.28  (23.63,24.94) | 23.08  (21.58,24.57) | 27.23  (24.86,29.60) | 10.09  (9.80,10.38) | 11.26  (10.94,11.58) |
| Q2 (160.2,167.3) | 20.84  (20.14,21.54) | 23.98  (23.09,24.87) | 21.91  (21.42,22.41) | 25.38  (24.64,26.11) | 20.86  (19.67,22.05) | 30.43  (28.08,32.79) | 10.50  (10.13,10.87) | 12.13  (11.75,12.50) |
| Q3 (167.3,174.8) | 25.11  (23.98,26.24) | 26.77  (25.58,27.96) | 24.12  (23.32,24.92) | 26.11  (25.19,27.02) | 25.23  (23.76,26.70) | 33.40  (29.96,36.84) | 11.62  (11.21,12.03) | 13.21  (12.85,13.57) |
| Q4 (174.8,204.1) | 29.61  (28.43,30.79) | 29.39  (28.15,30.64) | 26.19  (25.26,27.11) | 28.02  (27.00,29.05) | 29.47  (27.19,31.75) | 32.93  (31.10,34.76) | 12.80  (12.45,13.16) | 14.17  (13.80,14.54) |
| **Weight (Kg)** |  |  |  |  |  |  |  |  |
| Q1 (32.3,66.6) | 19.09  (18.25,19.93) | 21.69  (20.75,22.64) | 22.46  (21.66,23.26) | 25.51  (24.62,26.41) | 17.37  (16.32,18.43) | 26.44  (24.31,28.58) | 11.74  (11.38,12.11) | 12.23  (11.94,12.52) |
| Q2 (66.6,78.7) | 21.54  (20.72,22.36) | 24.57  (23.38,25.75) | 22.64  (21.93,23.36) | 25.84  (24.86,26.81) | 20.52  (19.20,21.84) | 29.88  (28.23,31.53) | 11.53  (11.10,11.96) | 12.83  (12.42,13.23) |
| Q3 (78.7,92.9) | 24.89  (24.04,25.73) | 27.97  (26.98,28.96) | 23.81  (23.00,24.62) | 26.82  (25.97,27.66) | 26.10  (24.80,27.40) | 32.38  (30.40,34.35) | 11.32  (10.92,11.72) | 13.52  (13.12,13.91) |
| Q4 (92.9,196.6) | 30.42  (29.17,31.68) | 29.83  (28.72,30.93) | 26.00  (24.98,27.01) | 26.67  (25.75,27.60) | 32.49  (30.43,34.55) | 36.59  (32.65,40.54) | 10.83  (10.52,11.14) | 13.01  (12.61,13.42) |
| **BMI** |  |  |  |  |  |  |  |  |
| Q1 (15.02,24.28) | 19.33  (18.66,19.99) | 23.10  (21.79,24.41) | 22.35  (21.80,22.91) | 26.50  (25.43,27.58) | 17.38  (16.32,18.44) | 27.44  (25.66,29.23) | 12.30  (11.88,12.72) | 13.15  (12.77,13.52) |
| Q2 (24.28,27.90) | 22.46  (21.46,23.45) | 25.52  (24.55,26.48) | 23.46  (22.56,24.36) | 26.41  (25.47,27.35) | 20.98  (19.87,22.10) | 30.70  (28.61,32.79) | 12.04  (11.58,12.51) | 13.09  (12.73,13.45) |
| Q3 (27.90,32.41) | 26.45  (25.46,27.44) | 28.04  (26.91,29.18) | 24.14  (23.13,25.14) | 26.33  (25.42,27.24) | 27.75  (25.88,29.62) | 31.98  (30.19,33.77) | 11.24  (10.90,11.58) | 13.15  (12.75,13.55) |
| Q4 (32.41,84.87) | 28.89  (27.67,30.10) | 28.25  (27.36,29.13) | 25.40  (24.40,26.41) | 25.52  (24.95,26.09) | 31.67  (29.76,33.58) | 36.62  (32.13,41.11) | 9.98  (9.67,10.30) | 12.11  (11.71,12.51) |

Table 2s. The coefficients of independent risks of MAFLD

|  | **Estimate** | **Std. Error** | **z value** | **Pr(>\|z\|)** |
| --- | --- | --- | --- | --- |
| **(Intercept)** | -8.80778 | 0.626596 | -14.057 | < 2e-16 |
| **Lead** | 0.140555 | 0.065303 | 2.152 | 0.031369 |
| **Diabetes** |  |  |  |  |
| IGT | Reference |  |  |  |
| IFG | 0.394449 | 0.187237 | 2.107 | 0.035145 |
| DM | 0.801287 | 0.185321 | 4.324 | 1.53E-05 |
| **Hypertensionyes** | 0.397927 | 0.141822 | 2.806 | 0.005019 |
| **Hyperlipidemiayes** | 0.38943 | 0.159932 | 2.435 | 0.014893 |
| **VAI** | 0.251445 | 0.060514 | 4.155 | 3.25E-05 |
| **BMI** | 0.07753 | 0.025562 | 3.033 | 0.002421 |
| **Ethnicity** |  |  |  |  |
| Non-Hispanic White | Reference |  |  |  |
| Waist circumference | 0.051868 | 0.010725 | 4.836 | 1.32E-06 |
| Mexican American | 0.48173 | 0.218942 | 2.2 | 0.027788 |
| Non-Hispanic Black | -0.6323 | 0.192089 | -3.292 | 0.000996 |
| Other Hispanic | -0.12975 | 0.25 | -0.519 | 0.603749 |
| Other Race - Including Multi-Racial | 0.005018 | 0.192471 | 0.026 | 0.979199 |
| **Education** |  |  |  |  |
| Less than high school | Reference |  |  |  |
| High school grad | 0.522976 | 0.210277 | 2.487 | 0.012879 |
| More than high school | 0.361215 | 0.187235 | 1.929 | 0.053705 |

IGT: impaired glucose tolerance; IFG: impaired fasting glucose; DM: diabetes mellitus
