# Supplementary figures and images for: Lead exposure as a causative factor for metabolic associated fatty liver disease (MAFLD) and a lead exposure related nomogram for MAFLD prevalence
Source: Front Public Health. 2022 Oct 12;10:1000403. doi: 10.3389/fpubh.2022.1000403 (PMC9597460; doi:10.3389/fpubh.2022.1000403)

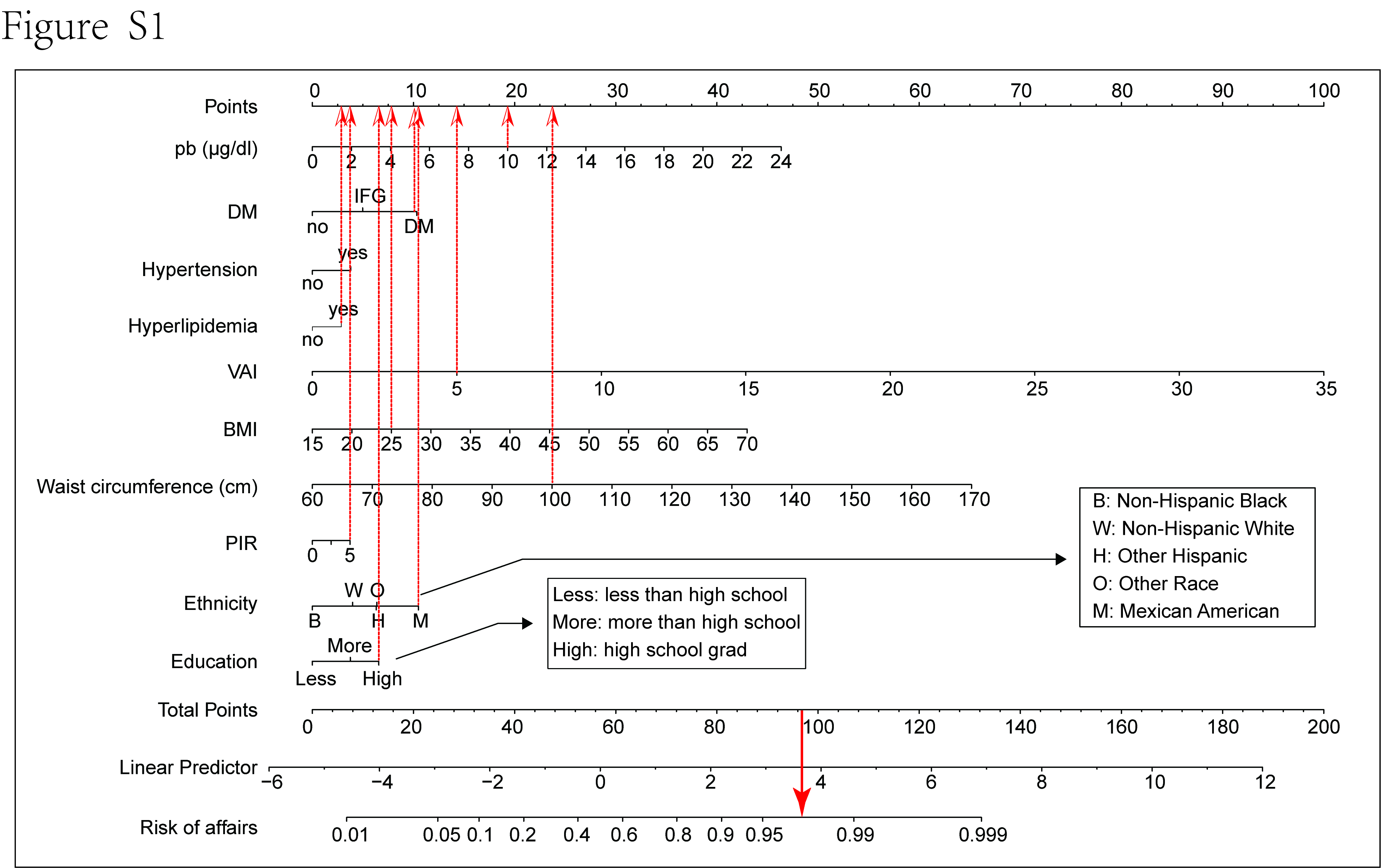

Supplement: Supplementary file 2 [file Image_1.TIF]
